# Supplementary material for: Substitution of Coxsackievirus A16 VP1 BC and EF Loop Altered the Protective Immune Responses in Chimera Enterovirus A71
Source: Vaccines (Basel). 2023 Aug 14;11(8):1363. doi: 10.3390/vaccines11081363 (PMC10458053; doi:10.3390/vaccines11081363)
Supplement: Supplementary file 1 [file vaccines-11-01363-s001.zip › vaccines-2505887-supplementary.pdf]

A

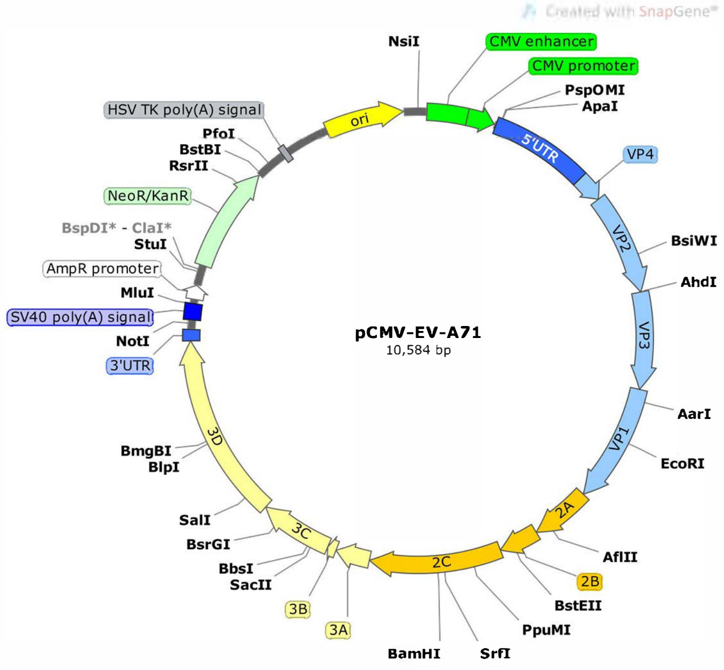

B

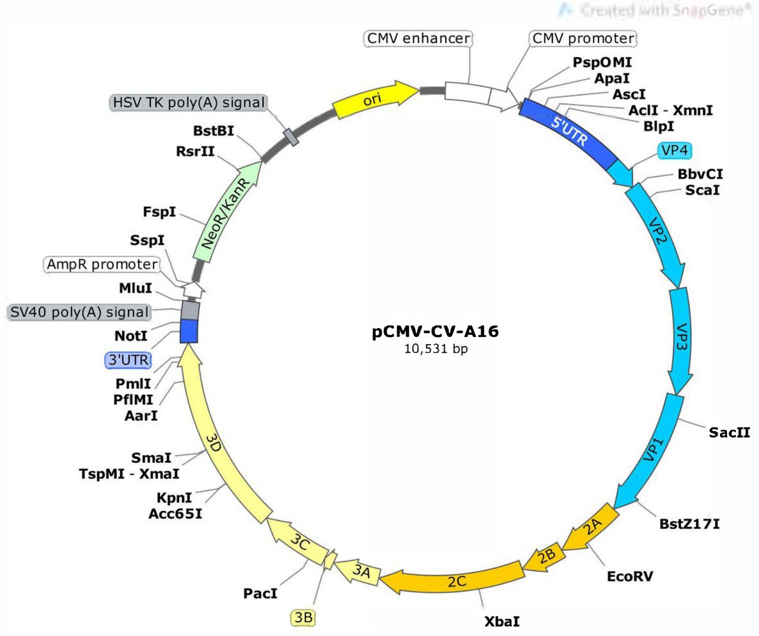

Supplementary Figure S1: Plasmid maps of EV-A71 (A) and CV-A16 (B) infectious clones used in this study.

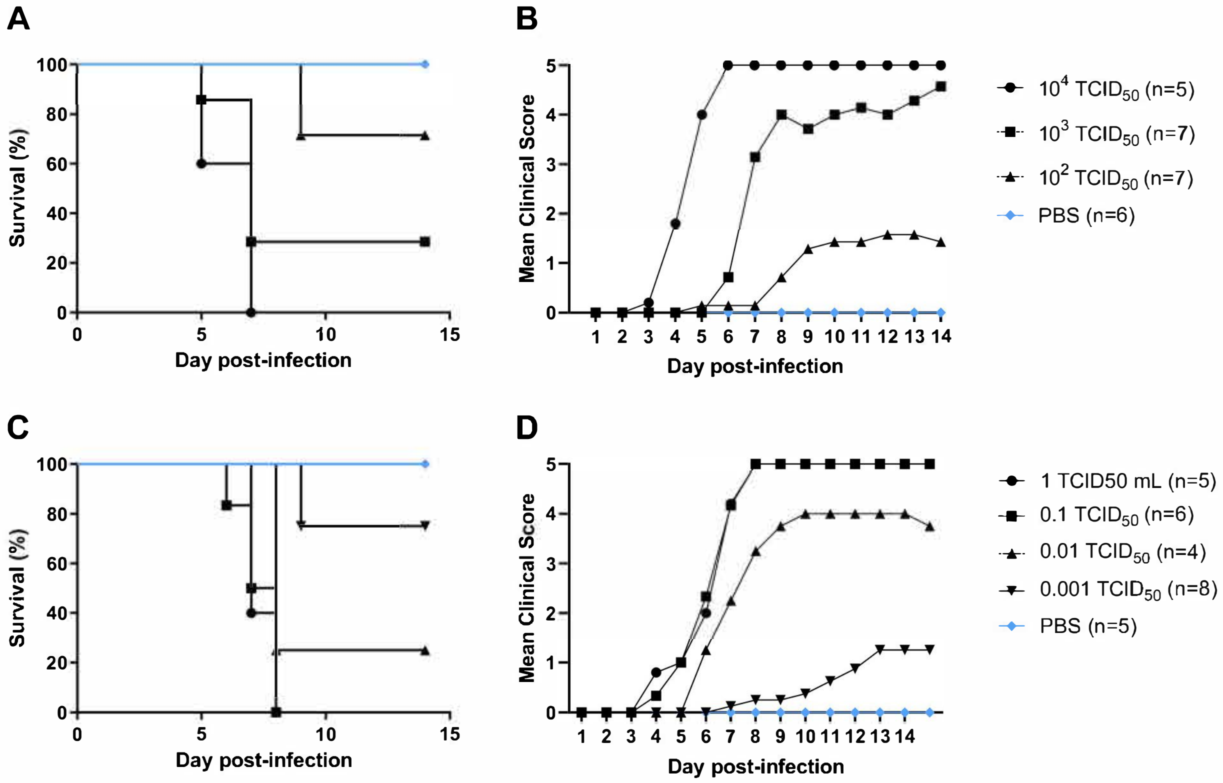

Supplementary Figure S2: LD_{50} determination of MP4 EV-A71 and WT CV-A16 in newborn mice. (A) Survival curve and (B) mean clinical score of mice inoculated with MP4 EV-A71. (C) Surviv-al curve and (D) mean clinical score of mice inoculated with CV-A16. The mice were monitored for 14 days for survival and clinical symptoms. Clinical scores were defined as: 0, healthy; 1, weak or less active; 2, hunched posture and lethargy; 3, one-limb paralysis; 4, two-limb paralysis; 5, moribund or dead.

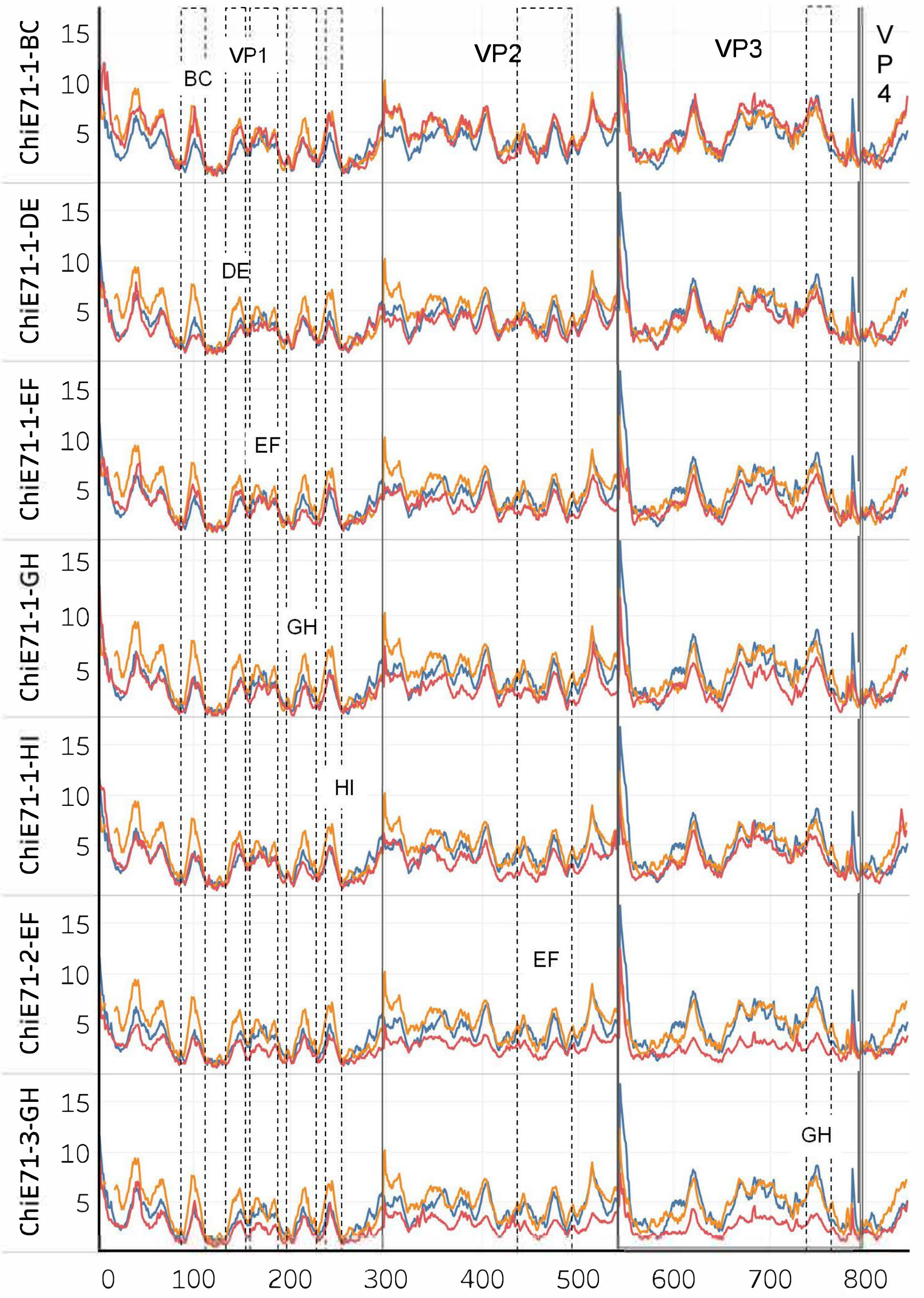

Supplementary Figure S3: Root mean square fluctuation (RMSF) of VP1 of ChiE71, WT EV-A 71 and WT CV-A16. Molecular dynamics (MD) simulations were performed using AMBER 20 with trajectory analysis using the CPPTRAJ module. The y-axis of each graph is the average RMSF of each residue during 10 ns MD simulation. ChiE71, WT EV-A71 and WT CV-A16 are depicted in red, blue and orange, respectively.

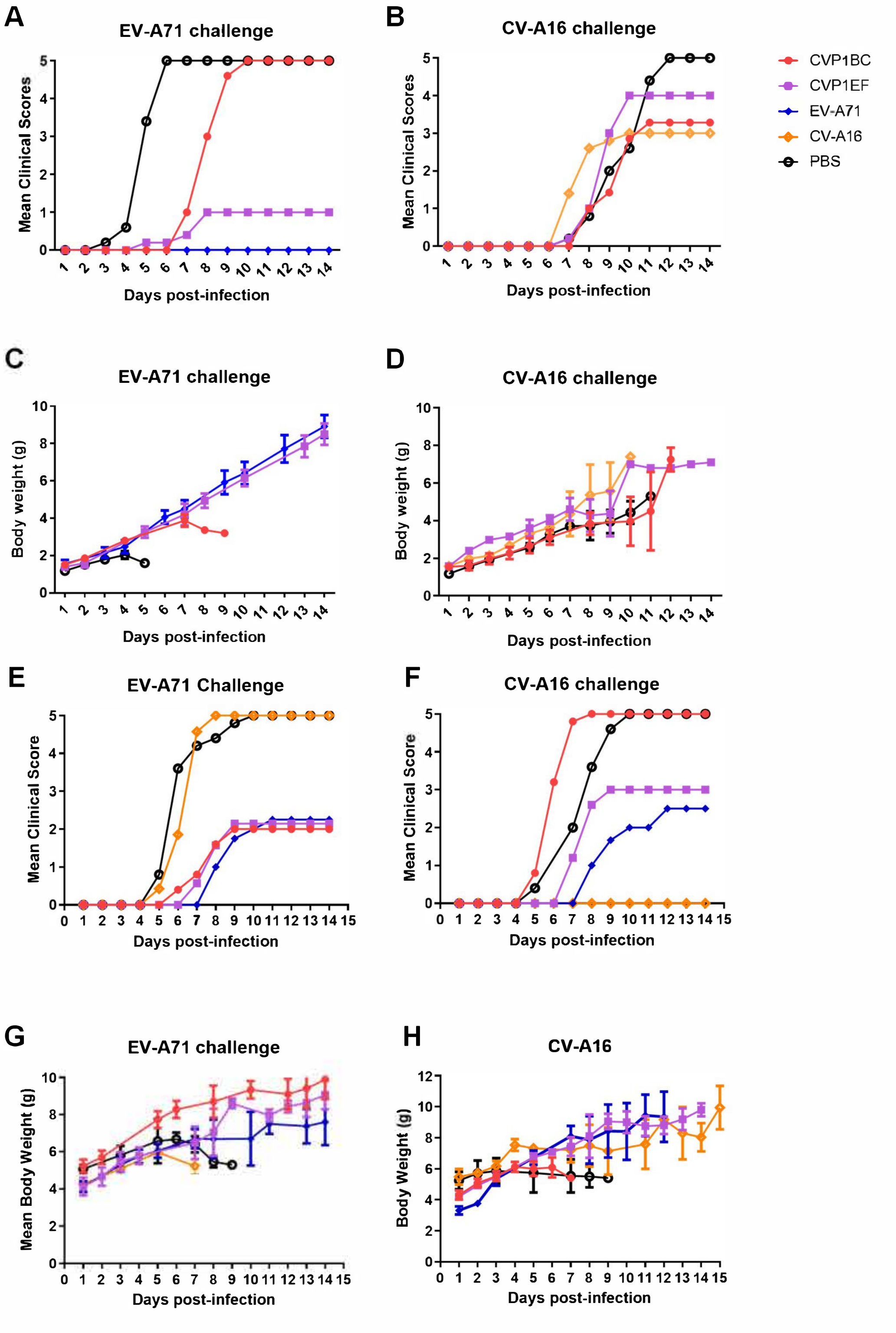

Supplementary Figure S4: Mean clinical scores and body weights of immunized neonatal mice subjected to virus challenge. (A and C) Mean clinical scores and body weights of immunized mice subjected to EV-A 71 challenge. (B and D) Mean clinical scores and body weights of passively immunized neonatal mice sub-jected to CV-A16 challenge. (E and G) Mean clinical scores and body weights of actively immunized mice subjected to EV-A71 challenge. (F and H) Mean clinical scores and body weights of actively immunized neonatal mice subjected to CV-A16 challenge. Error bars represent the SDs of each group at each time point.

Table S1. Primer pairs used for site directed mutagenesis.

Primer Set	Primer Code	Nucleotide Sequence (5′→3′)	Target Region	Product size (base)	First Annealing Temperature (°C)	Second Annealing Temperature (°C)	Polarity
**ChiE71-1-BC**	1BC F	ACACAGATGGTTACGTTAATTGGGATATAGA CATAACTGGTTACGC	VP1 BC loop	89	67	72	Sense
	1BC R	TCTGTGTACCCGTGGTGGGGAGATCTATCTC TCCTACCAAGCC					Antisense
**ChiE71-1-DE**	1DE F	AGTCCCCCAATTACTTCAGTATATGTTTGTTCC	VP1 DE loop	72	58	70	Sense
	1DE R	AGCTCACCATTGGGTTTGCACGCAACAAAAGT GAATTCC					Antisense
**ChiE71-1-EF**	1EF F	GATTCATTCGCTTGGCAAACTGCTACCAACCC ATCAGTTTTTGTCAAGTTGACTGATCCC	VP1 EF loop	117	64	72	Sense
	1EF R	TCTGGAAGTGGGTTTCGGAGCCCCTGGCGGAA CAAACATATACTGAAGTAATTGTG GG					Antisense
**ChiE71-1-HI**	1HI F	AGTCCCACACTCCAAGGATATATATGAGAATG AAGC	VP1 HI loop	76	57	72	Sense
	1HI R	TCTCGGTCCCTACTCGCACCGAGAAAGTGCCC ATCATATTATT					Antisense
**ChiE71-3-GH**	3GH F	CGTGCTGGGTACTTTGACTATTACACCACCGG CCTGGTTAGTATCTGGTATCAAACAAAC	VP3 GH loop	120	63	72	Sense
	3GH R	GGCATGCGCCCTATAGTGTGTGTTACTAATCC ATGGTATTACAAGGGTGACAGATGATTG					Antisense
**ChiE71-1-1GH**	1GH F	ATCCCCCGGCACAGGTCTCAGTTCCCTTCATGT CACCAGCC	VP1 GH loop	41	72	72	Sense
	1GH R	AAAGTGCCCATCATATTATTCGGACATTGACCA TAATCTAGG		42	72	72	Antisense
	1GH F2	DNA product of PCR using 1GH F and 1GH R:		145	65	72	Sense

		ATCCCCCGGCACAGGTCTCAGTTCCCTTCATGT CACCAGCCAGCGCATACCAGTGGTTTTATGATG GTTATCCCACCTTTGGAGAACATCTCCAAGCAA ATGACCTAGATTATGGTCAATGTCCGAAT AATATGATGGGCACTTT					
	1GH R2	CAGTCAACTTGACAAAAACTGAGG		24	65	72	Antisnese
**ChiE71-2-EF**	CVP2 EF F	TATGTGCTCGGCACTATCGC	VP2 EF loop	20	68	72	Sense
	CVP2 EF R1	TCCGTAGGTTAATCCATTGGTGAGGGCATA CAGTTAATTGGCTCAAAGGG		50	68	72	Antisense
	CVP2 EF F	DNA product of PCR using CVP2 EF F and CVP2 EF R: TATGTGCTCGGCACTATCGCAGGAGGGACC GGGAACGAGAATTCTCATCCCCCCTACGCC ACTACACAGCCTGGTCAGGTTGGTGCAGTC CTGACGCACCCTTATGTGCTAGATGCAGGG ATCCCTTTGAGCCAATTAACTGTATGCCCTC ACCAATGGATTAACCTACGGA		172	65	72	Sense
	CVP2 EF R2	CTCTGGAAGTATAGCGACTAACAACGCTCCT TGATGGAACTTGC		44	65	72	Antisense
